# Essential tremor-challenged maxillary rehabilitation using a digitally guided all-on-six implant restoration: a case report

**DOI:** 10.3389/froh.2025.1663892

**Published:** 2025-09-12

**Authors:** Shuning Zhang, Jiarui Cui, Fan He, Jiani Hu, Guangzheng Yang, Hui Huang, Jie Wang, Xinquan Jiang

**Affiliations:** 1 Department of Prosthodontics, Shanghai Ninth People’s Hospital, Shanghai Jiao Tong University School of Medicine, Shanghai, China; 2 Department of Prosthodontical Technique, Shanghai Ninth People’s Hospital, Shanghai Jiao Tong University School of Medicine, Shanghai, China; 3 Shanghai Stomatological Hospital, Fudan University, Shanghai, China

**Keywords:** elderly edentulism, all-on-six, essential tremor, digital workflow, digital jaw relation transfer, case report

## Abstract

This case report describes a 73-year-old female with essential tremor who experienced significant dissatisfaction with her maxillary complete denture. Her tremor-related dexterity impairment limited her ability to manage removable prostheses, while the excessive denture volume severely disrupted speech. Moreover, her neuromuscular condition made it challenging to adapt to a new occlusal scheme. To address these issues, a digitally guided all-on-six implant-supported prosthesis was delivered under local anesthesia, preserving her pre-existing, neuromuscularly adapted occlusion with minor esthetic adjustments to the anterior teeth. At the 1-year follow-up, the patient reported high satisfaction with both esthetics and function, along with improved speech and masticatory efficiency. This report underscores the value of digital workflows in maintaining functional adaptation and providing predictable prosthodontic solutions for patients with neuromuscular impairments.

## Introduction

Essential tremor is a common movement disorder in elderly, often characterized by rhythmic, involuntary oscillations of the upper extremities, head, face and tongue (1, 2). The tremors present specific challenges to prosthodontic rehabilitation by impairing manual dexterity, denture retention, and neuromuscular adaptation (3). Conventional removable prostheses are often unsatisfactory in this population due to limited oral control, reduced prosthesis stability, and discomfort during insertion or removal (4, 5).

Implant-supported fixed prostheses can improve function, retention, and quality of life in edentulous patients (6). However, in tremor-affected individuals, success may be limited by difficulties in recording accurate occlusal relationships and achieving postoperative neuromuscular adaptation (7–11). In addition, intraoperative tremors can interfere with guided surgery and the insertion of prosthesis (12).

Digital dentistry provides opportunities to streamline treatment workflows and improve prosthetic accuracy (13, 14). The integration of intraoral scans, cone beam computed tomography (CBCT), and existing prostheses can support prosthetically driven implant planning while preserving the patient's adapted occlusal scheme (15, 16). In patients with tremor, this approach may reduce the need for repeated functional reprogramming and reduce chair time.

This case report presents the rehabilitation of a 73-year-old edentulous patient with a 20-year history of essential tremor, manifesting as bilateral upper limb tremors along with tremors in the jaw and tongue. A digitally guided all-on-six implant-supported maxillary prosthesis was fabricated by replicating the occlusal relationship of her existing complete denture (CD). A delayed loading protocol was employed to accommodate age-related healing limitations (17, 18). At the 1-year follow-up, the patient exhibited stable function and high satisfaction by T-scan findings and OHIP-EDENT scores (19, 20). This report highlights the potential of digital workflows in the prosthodontic management of patients with neurological movement disorders.

## Case report

**Figure 1:**
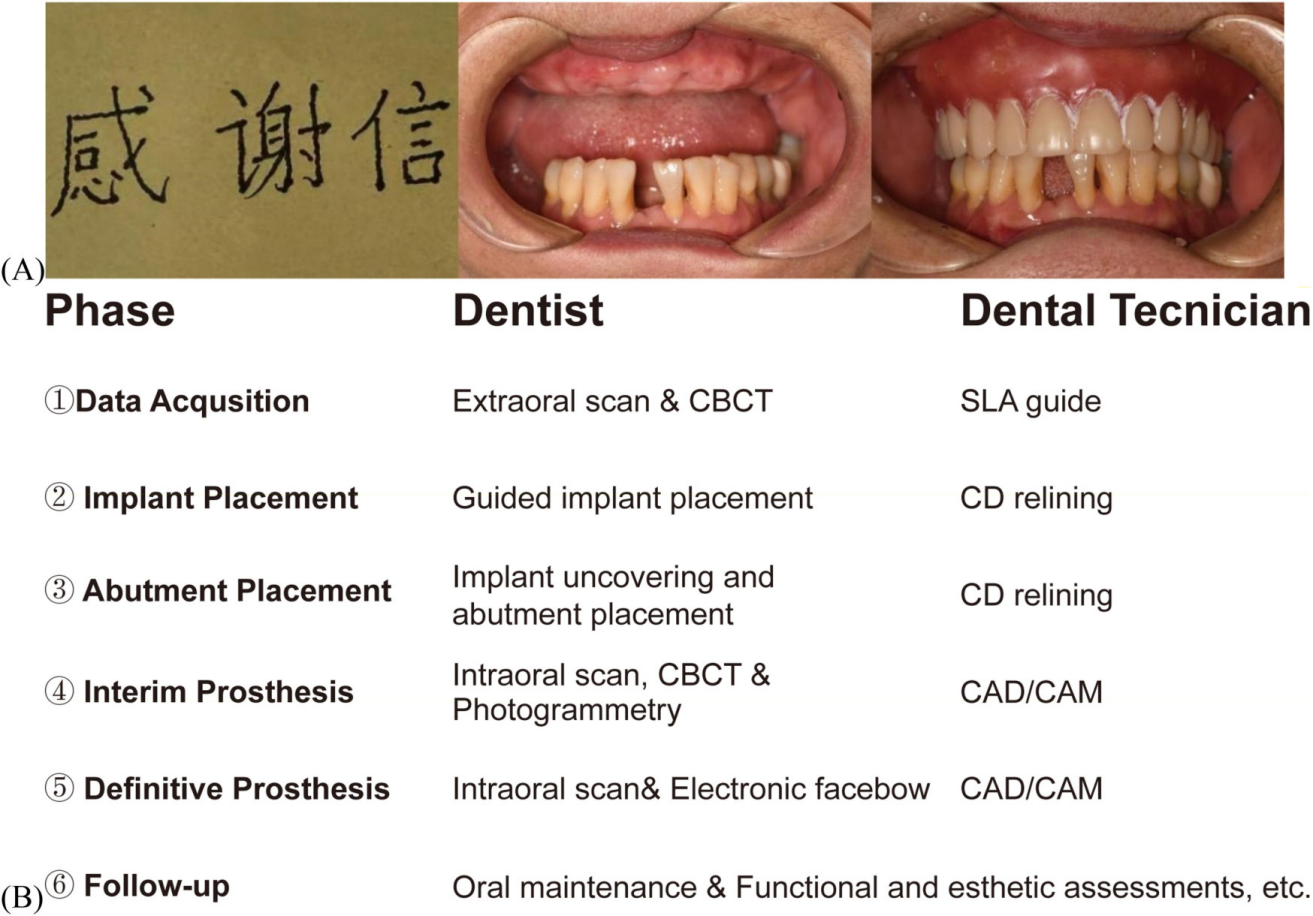
Cinical photographs and digital treatment plan of patient. **(A)** Patient's handwriting and intraoral images, including one with a CD. **(B)** Digital workflow for maxillary all-on-six prostheses in tremor patients: clinician-technician synergy.

A 73-year-old female patient presented with a 20-year history of essential tremor, demonstrating familial predisposition as her younger brother was similarly affected. Neurological examination confirmed bilateral upper limb postural and kinetic tremors, predominantly exacerbated during voluntary actions and emotional stress, consistent with criteria for essential tremor according to the 2018 International Parkinson and Movement Disorder Society guidelines (21). The tremor phenotype also involved selective tremors in the lingual and jaw (Supplementary Video S1), along with a tremulous handwriting pattern (Figure 1A). The patient had trialed first-line agents, including propranolol, gabapentin, arotinolol, and primidone, but discontinued due to dose-related hypotension.In September 2023, the patient underwent extraction of all upper teeth, leaving only a high-position impacted tooth #28, and presenting as a completely edentulous maxillary arch (Figure 1A). In the mandible, an implant had previously been placed for tooth #36 at another clinic. Teeth #37, #41, #46, and #47 remained unrestored, and extraction was planned for tooth #48. In November 2023, the patient's CD was provided by an experienced physician (Figure 1A). The patient was satisfied with the occlusal relationship but reported impaired speech and difficulty in CD removal, attributed to tremor-related oral dysfunction. For this reason, we discussed with the patient and decided to change to a fixed implant-supported restoration. To facilitate the transition from CD to a fixed restoration, a fully digital workflow was designed and implemented (Figure 1B).

**Figure 2:**
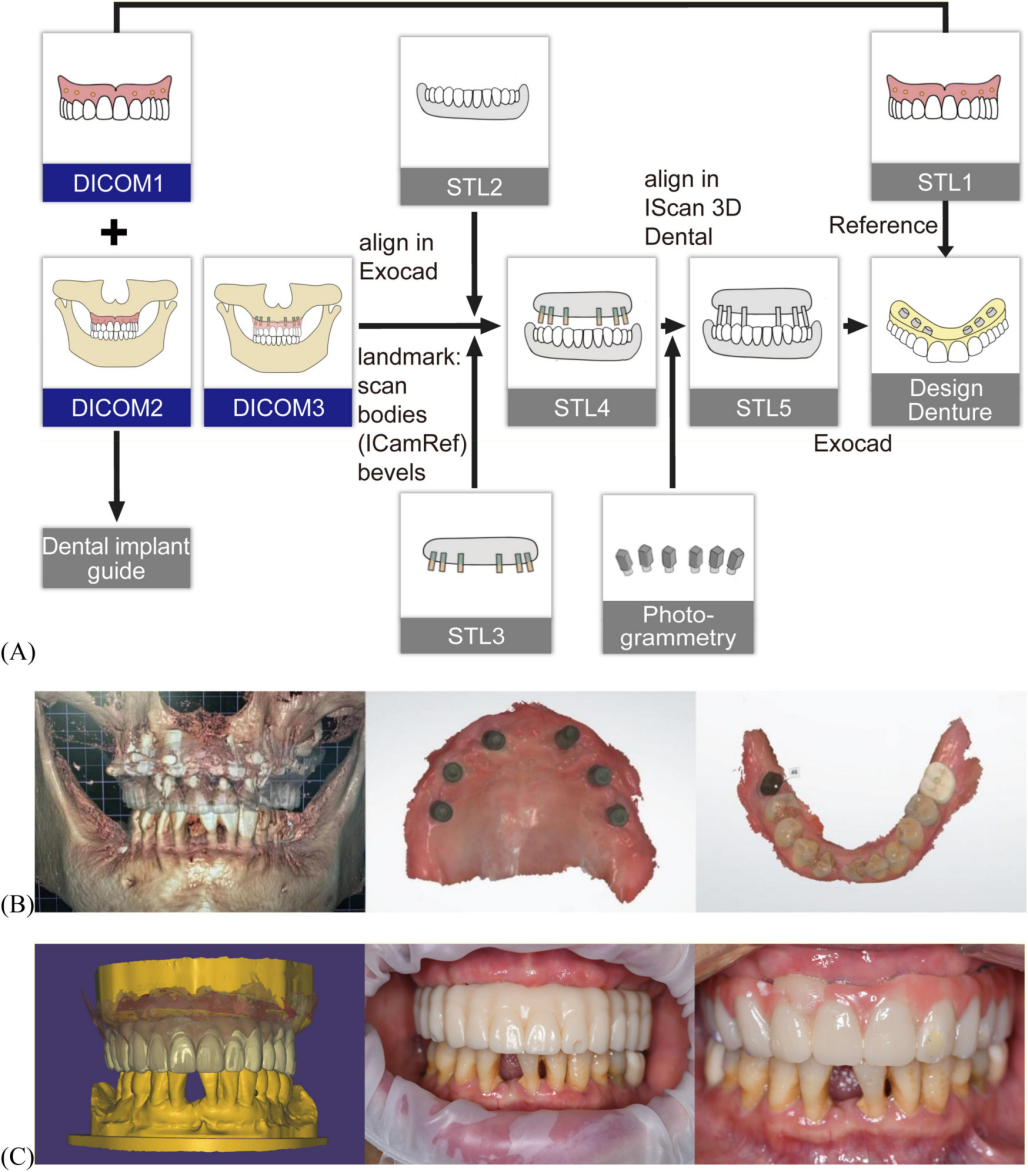
Digital workflow for maxillomandibular relationship transfer and prosthetic design. **(A)** Schematic illustration of digital workflow for maxillomandibular relationship transfer and prosthetic design. Maxillary CD CBCT scan—data as DICOM1, maxillary CD scan—data as STL1, patient wearing maxillary CD in centric occlusion during CBCT scan—data as DICOM2, DICOM1 and DICOM2 were imported into 3Shape Implant Studio™ for surgical guide design. The maxillary CD was relined at the implant healing cap sites and another CBCT was performed with the CD in centric occlusion—data as DICOM3, mandibular intraoral scan—data as STL2; maxillary intraoral scan with healing caps—data as STL3, STL2 and STL3 were aligned using DICOM3 to generate STL4, ICAM 4D photogrammetry was used to capture the maxillary implant positions, which were merged with STL4 to create STL5, the interim prosthesis was designed on STL5, with tooth arrangement referenced from STL1. **(B)** Postoperative CBCT, intraoral scan of the maxilla and mandible. **(C)** Digital design of the prosthesis, intraoral view of temporary resin prosthesis and interim prosthesis.

**Figure 3:**
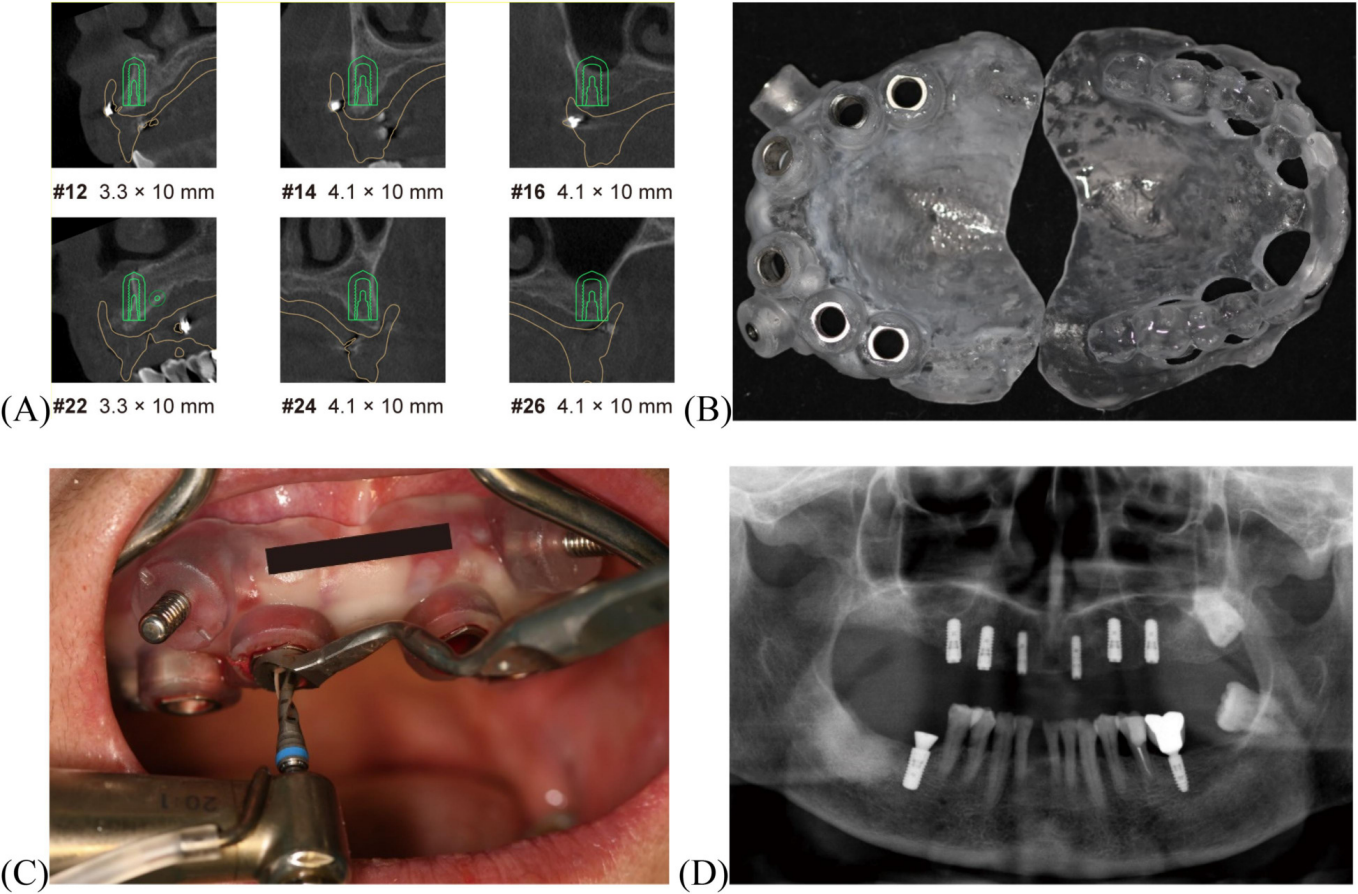
Digitally guided implant procedure. **(A)** CT image showing implant planning. **(B)** Implant surgical guide with occlusal registration. **(C)** Implant placement with guide. **(D)** Postoperative panoramic radiograph.

Eight concave depressions were prepared on the tissue surface of the existing CD. These depressions were packed with gutta-percha to create a radiographic guide. A CBCT (I-Cat, Imaging Sciences International, Pennsylvania, USA) scan was first acquired of the radiographic guide alone (Figure 2A DICOM1). The guide was then seated intraorally in centric occlusion, and a second CBCT scan was obtained (Figure 2A DICOM2). Both CBCT datasets were imported into 3Shape Implant Studio™ software. Prosthetically driven planning for maxillary implant placement (Figure 3A) and surgical guide design was performed, resulting in a surgical guide that preserved the existing occlusal relationship. The prosthetically driven implant surgical stackable guides with occlusal registration were printed (Figure 3B and Supplementary Figure S1) (22). In clinical use, the double-layer guide is placed intraorally and the patient bites in centric occlusion (Supplementary Figure S1). After fixing the surgical guide with fixation pins, the occlusal guide is removed, exposing the designed implant guide holes for precise implant placement. Despite challenges posed by involuntary tremors and limited interocclusal space in the patient, the digitally guided protocol ensured precise positioning of the implants within 40 min, performed under local infiltration anesthesia. The implants used were ITI RC BL with SLA surface treatment. Implant sites included #12 and #22 [3.3 × 10 mm], #14, #24, #16, and #26 [4.1 × 10 mm], and tooth #46 was concurrently implanted [4.8 × 10 mm] (Figure 3C and Figure 3D). Primary stability was achieved at the time of placement, with insertion torque values between 10 and 15 N · cm. A submerged (two-stage) healing protocol was chosen to optimize osseointegration, considering the patient's advanced age and female gender. At the four-month follow-up, the Implant Stability Quotient (ISQ) was measured (Supplementary Table S1) and transmucosal healing caps and multiunit abutments were used. The original CD was relined and adjusted to support occlusal function during implant osseointegration.

To replicate the patient's adapted occlusal relationship, a digital workflow for occlusion planning was applied (Figure 2A). The maxillomandibular relationship was transferred using CBCT, intraoral scanning (3shape Trios 3 Pod, 3Shape, Copenhagen, Denmark), and photogrammetry (ICam4D, IMetrics4D Imaging, Courgenay, Switzerland) data (Figure 2B). Based on the integration of these datasets, a temporary prosthesis was digitally fabricated using a CAD/CAM workflow according to the implant positions in STL 5 and the tooth arrangement of the CD in STL 1, replicating the functional occlusion (Figure 2C left). As the patient reported that the palatal contour of the anterior teeth in the CD affected speech, localized modifications were made to the anterior teeth (#2-2). These adjustments involved a slight overall labial repositioning and minor lingual inclination of the incisal edges. The contour and position were trialed using a temporary resin prosthesis (Figure 2C middle). Following patient communication and intraoral adjustments, the patient expressed satisfaction. The new anterior prosthetic morphology and position were then adopted for the aluminum prosthesis (the interim prosthesis, Figure 2C right). Oral hygiene instructions included the use of an electric toothbrush and regular follow-up visits for oral health assessment and maintenance.

**Figure 4:**
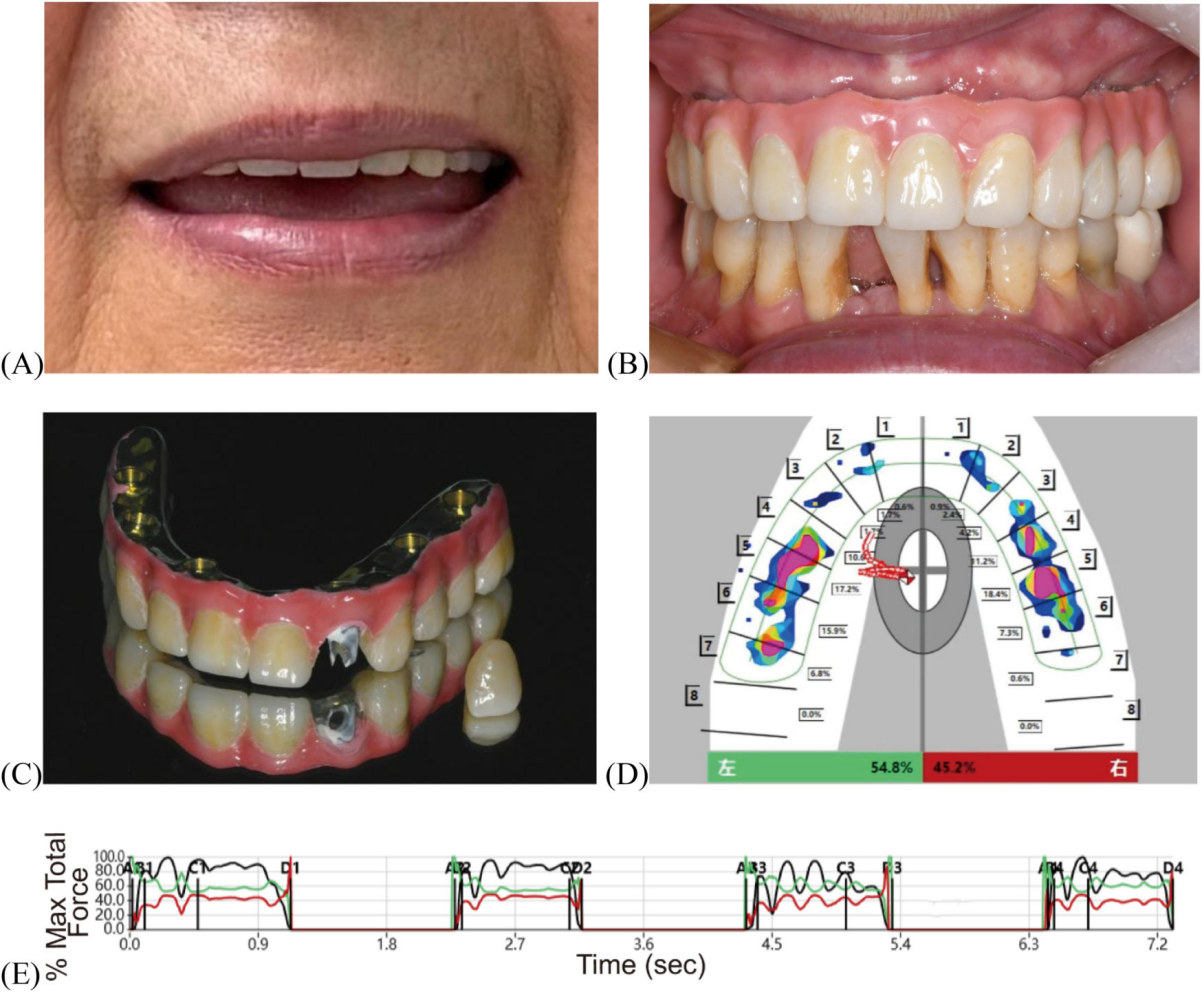
Post-treatment evaluation of definitive prosthesis. **(A)** Facial photograph. **(B)** Intraoral view. **(C)** Final restoration. **(D)** T-Scan mapping. **(E)** T-Scan analysis.

Following a three-month adaptation period for maxillomandibular relation and occlusion using the interim prosthesis, intraoral scans of the prosthesis *in situ*, the mucosa (after prosthesis removal), the opposing arch, and the occlusal registration were acquired. This scan data was then merged with the previously obtained ICAM 4D dataset. During the process, occlusal wear patterns from the interim prosthesis were integrated via Boolean operations to refine the occlusal scheme.The definitive prosthesis was fabricated using CAD/CAM technology. It consisted of an anodized titanium framework supporting monolithic zirconia crowns (Lava Ultimate, 3M ESPE, USA) with the gingival portion fabricated in multi-shade light-curing composite resin material (Shofu Dental, Japan) (Figures 4A,B). The passivity of the titanium framework was verified intraorally by the chief clinician using “alternate finger pressure” method and probe testing to ensure a passive fit (23). The metal framework was treated by sandblasting and gold plating to enhance bonding strength, and then the crowns were cemented using 3M™ RelyX™ U200 adhesive. To address the labial screw access at site #22, a retrievable single crown was designed without compromising aesthetics (Figure 4C). At the one-year postoperative follow-up, T-Scan analysis demonstrated occlusion time <0.2 s and midline-aligned center of force, confirming clinically occlusal stability (Figures 4D,E; Supplementary Tables S2, S3) (24). Post-treatment OHIP-EDENT scores showed a significant reduction, from 57 to 22 in the total score (Supplementary Table S4), with only mild residual speech adaptation issues (19, 20). The patient reported greater ease in daily oral hygiene maintenance and noted that cleaning the prosthesis became simpler and more efficient. She also described feeling more confident when chewing, with improved masticatory strength and no discomfort during meals. Overall, she expressed high satisfaction with the final prosthesis and described it as comfortable and stable in daily activities.

## Discussion

In this case report, a digitally guided all-on-six protocol was used to restore a 73-year-old edentulous patient with essential tremor. Implant planning was based on her existing CD, which had provided a functionally adapted occlusal relationship. Digital duplication of the denture preserved this relationship, reducing the need for occlusal reprogramming. A delayed loading protocol was chosen due to the patient's age and limited healing capacity (17, 18).

The digital workflow enabled precise implant positioning under local anesthesia using a stereolithographic guide, avoiding general anesthesia often required in tremor cases (12). An interim prosthesis phase allowed for stepwise occlusal refinement before definitive restoration. The definitive prosthesis accurately replicated the patient's adapted occlusion, with anterior modifications to enhance esthetics and phonetics. At the 1-year follow-up, the prosthesis remained stable without complications. The patient reported improved speech and mastication, highlighting the benefit of minimizing intraoral bulk in individuals affected by tremor.

An important consideration in the treatment plan was the choice between four vs. six implants to support the maxillary CD. Although both all-on-four and all-on-six approaches can achieve satisfactory function and prosthesis survival rates, the all-on-six protocol provides greater biomechanical stability and more favorable force distribution, especially in elderly patients or those with complex conditions such as bruxism (25, 26). In this patient, the all-on-six design not only increased the anterior-posterior spread of the implants, enhancing prosthesis support and retention. This choice was especially prudent given the patient's age-related bone resorption and tremor condition.

Furthermore, essential tremor and upper limb tremors present additional challenges for peri-implant oral hygiene maintenance. Tremor-related manual dexterity impairment often leads to difficulties in oral cleaning routine (27). For patients with hand tremors, using an electric toothbrush in combination with water flossers and mouthwash is an appropriate approach to maintaining oral health (28, 29). In our case, the fixed implant-supported restoration was designed with a contour that allowed easy access for cleaning devices. Compared to removable dentures, which required repeated insertion and removal and often accumulated food debris in undercuts, the fixed prosthesis was reported by the patient to be significantly easier and faster to clean. As a result, the patient subjectively perceived daily oral hygiene to be more convenient, less fatiguing, and more thorough. However, there is currently a lack of a widely recognized, evidence-based care protocol specifically for fixed implant prostheses in patients with neurological disorders. As idiopathic tremor progresses in such patients, their oral hygiene maintenance model needs to shift from a “patient-centered” to a “caregiver-centered” approach. This transition is essential to ensure that these patients with special needs can benefit from modern dental restorations while also maintaining long-term oral health and quality of life (30).

Nevertheless, a major limitation of this report is the one-year follow-up duration, which may not fully capture potential long-term complications or maintenance challenges. Further longitudinal studies with extended follow-up are necessary to validate these promising outcomes.

This report demonstrates that replicating a neuromuscularly adapted denture through digital workflows may improve predictability and reduce adaptation demands in complex prosthodontic cases. The use of fixed implant-supported restorations in tremor patients is not only feasible but also offers significant advantages in terms of retention, stability, and overall functionality compared to conventional removable prostheses (6). While promising, this approach relies on precise denture adaptation and clinical expertise. Further studies are needed to evaluate its long-term outcomes in patients with movement disorders.

## Summary

Digitally guided all-on-six rehabilitation offered a predictable solution for restoring function and esthetics in a tremor-affected elderly patient by replicating an existing, neuromuscularly adapted occlusal relationship. This approach may represent a viable treatment option for similar patients facing movement-related prosthodontic challenges.

## Data Availability

The raw data supporting the conclusions of this article will be made available by the authors, without undue reservation.
