# Supplementary material for: Essential tremor-challenged maxillary rehabilitation using a digitally guided all-on-six implant restoration: a case report
Source: Front Oral Health. 2025 Sep 12;6:1663892. doi: 10.3389/froh.2025.1663892 (PMC12463972; doi:10.3389/froh.2025.1663892)
Supplement: Supplementary file 1 [file Datasheet1.docx]

Supplementary Material

# Supplementary Tables

**TABLE S1**

The Implant Stability Quotient of the implants, four months after the surgery

| Implant Stability Quotient | | | | | | |
| --- | --- | --- | --- | --- | --- | --- |
| Maxillary lateral incisor, right | Maxillary  first premolar, right | Maxillary first molar, right | Maxillary central incisor, left | Maxillary  first premolar, left | Maxillary first molar, left | Mandibular first molar, right |
| 73 | 69 | 75 | 73 | 60 | 82 | 78 |

**TABLE S2**

Occlusal force percentage distribution in definitive prosthesis

|  | Total | Central incisor | Lateral incisor | Canine | First premolar | Second premolar | First molar | Second molar |
| --- | --- | --- | --- | --- | --- | --- | --- | --- |
| Left | 54.8% | 0.6% | 1.7% | 1.7% | 10.6% | 17.2% | 15.9% | 6.8% |
| Right | 45.2% | 0.9% | 2.4% | 4.2% | 11.2% | 18.4% | 7.3% | 0.6% |

**TABLE S3**

Occlusion time

| Occlusion time | | | |
| --- | --- | --- | --- |
| OT1 | OT2 | OT3 | OT4 |
| 0.09 s | 0.05 s | 0.08 s | 0.04 s |

**TABLE S4**

OHIP-EDENT

| In the past one month | Score  Before | Score  After | In the past one month | Score  Before | Score  After |
| --- | --- | --- | --- | --- | --- |
| Q1 Prosthesis not fitting properly | 5 | 3 | Q11 Had to interrupt meals | 5 | 1 |
| Q2 Food stuck under the prosthesis | 3 | 1 | Q12 Unable to eat with prosthesis | 1 | 1 |
| Q3 Difficulty in chewing | 5 | 1 | Q13 Feeling uncomfortable | 1 | 1 |
| Q4 Painful aching in mouth | 4 | 1 | Q14 Being embarrassed | 2 | 1 |
| Q5 Uncomfortable to eat | 5 | 1 | Q15 Lack of satisfaction with friends and family | 1 | 1 |
| Q6 Mouth ulcer | 4 | 1 | Q16 Irritability with others | 1 | 1 |
| Q7 Uncomfortable prosthesis | 5 | 1 | Q17 Less focused with family | 1 | 1 |
| Q8 Self-conscious | 2 | 1 | Q18 Felt life less satisfying | 5 | 2 |
| Q9 Anxious | 2 | 1 | Q19 Avoid leaving the house | 1 | 1 |
| Q10 Avoid eating certain foods | 4 | 1 | Total score | 57 | 22 |
