# Supplementary material for: Essential tremor-challenged maxillary rehabilitation using a digitally guided all-on-six implant restoration: a case report
Source: Front Oral Health. 2025 Sep 12;6:1663892. doi: 10.3389/froh.2025.1663892 (PMC12463972; doi:10.3389/froh.2025.1663892)
Supplement: Supplementary file 2 [file Image1.pdf]

frontiers

Supplementary Material

1 Supplementary Figure

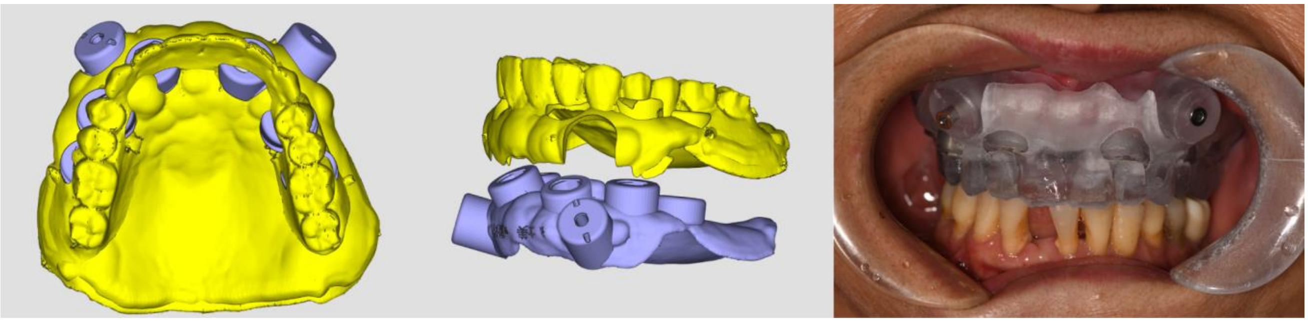

FIGURE S1

Design of the surgical guide (3Shape Implant Studio™ software) and intraoral try-in photograph.
